# The Trend in Distribution of Q223R Mutation of Leptin Receptor Gene in Amoebic Liver Abscess Patients from North India: A Prospective Study

**DOI:** 10.1155/2014/847132

**Published:** 2014-07-09

**Authors:** Anil Kumar Verma, Vineet Ahuja, Jaishree Paul

**Affiliations:** ^1^School of Life Sciences, Jawaharlal Nehru University, New Delhi 110067, India; ^2^Department of Gastroenterology, All India Institute of Medical Sciences, New Delhi 110052, India

## Abstract

Host genetic susceptibility is an important risk factor in infectious diseases. We explored the distribution of Q223R mutation in leptin receptor gene of amoebic liver abscess (ALA) patients of North India. A total of 55 ALA samples along with 102 controls were subjected to PCR-RFLP analysis. The frequency of allele “G” (coding for arginine) was in general high in Indian population irrespective of the disease. Our results of Fisher exact test shows that heterozygous mutant (QQ versus QR, *P* = 0.049) and homozygous mutant (QQ versus RR, *P* = 0.004) were significantly associated with amoebic liver abscess when compared with homozygous wild (QQ).

## 1. Introduction

The protozoan parasite* Entamoeba histolytica* is estimated to cause 100 million infections and 100,000 deaths worldwide annually and malnutrition is known to increase susceptibility to infection [1]. Amebiasis is more common in malnourished children, a state that afflicts approximately one-third of children in the developing world [2]. The most common manifestation of* E. histolytica* infection in India is either diarrhea with ulcerative lesion in caecum and colon (intestinal amebiasis) or amoebic liver abscess (ALA). Invasive trophozoites gain access to liver via hepatic portal vein and cause amoebic liver abscess due to necrotic lysis of the liver tissue. It varies in size and number. Necrotic lesions may be single or multiple and may occur in left or right lobe of liver. The common symptoms of ALA are fever, pain in the right hypochondrium, and liver tenderness [3, 4]. Recently a point mutation (Q223R, rs1137101, A to G) in leptin receptor gene at 223aa position has been found to be associated with the susceptibility to* E. histolytica* infection and disease outcome in Bangladesh [5]. It was observed that the children with allele for arginine (223R) were almost 4 times more susceptible to infection compared to those homozygous for the ancestral glutamine allele (223Q). In terms of nucleotides, Q (glutamine) is encoded by A (adenine) whereas R (arginine) is encoded by G (guanine). Leptin is a hormone/cytokine produced largely by adipocytes and to some extent by the stomach, skeleton muscles, and placenta [6]. Leptin plays several important roles in human physiology. It acts through the leptin receptor (LEP-R), a single-transmembrane-domain receptor of the cytokine receptor family which connects nutrition and immunity. In addition to regulating neuroendocrine function, energy homeostasis, haematopoiesis, and angiogenesis, leptin is an important modulator of both the innate and adaptive immune systems [7]. It has been earlier stated that malnutrition in children aggravates the propensity of amoebiasis [8]. Therefore, we proposed to study the segregation of alleles A and G of* LEPR* gene responsible for Q223R mutation in North Indian population and if it has any association with ALA.

## 2. Methodology

### 2.1. Biological Samples

The study patients were recruited from the Department of Gastroenterology, All India Institute of Medical Sciences, New Delhi, India, after necessary ethical clearances were obtained for the study. All the participating patients gave written consent for the study. ALA pus samples were collected from patients by the attending physician and necessary precautions were taken during pus aspiration from liver, so as to avoid any contamination. The pus aspiration from liver was carried out in those patients who did not respond to chemotherapy and where aspiration was unavoidable for case management. The demographic and clinical details of the patients are represented in Table 1. The samples were transported at 4°C within two hours after collection and stored at –20°C until processed. Blood samples from 102 individuals without any enteric or liver disease were included in the study as controls. The blood samples were collected by venipuncture in vacutainer tubes (BD NJ, USA) containing anticoagulant K_2_EDTA solution from individuals visiting the hospital.

### 2.2. DNA Extraction from Whole Blood and ALA Pus

DNA from blood samples was extractedusing standard protocol [9]. Briefly blood (stored in ACD or EDTA) was resuspended in 15 mL polypropylene centrifugation tubes with 3 mL of nuclei lysis buffer (10 mM Tris-HCl, 400 mM NaCl, and 2 mM Na_2_EDTA, pH 8.2). The cell lysates were digested overnight at 37°C with 0.2 mL of 10% SDS and 0.5 mL of a proteinase K solution (1 mg proteinase K in 1% SDS and 2 mM Na_2_EDTA). After digestion was complete, 1 mL of saturated NaCl (approximately 6 M) was added to each tube and shaken vigorously for 15 seconds, followed by centrifugation at 2500 rpm for 15 minutes. The precipitated protein pellet was left at the bottom of the tube and the supernatant containing the DNA was transferred to another 15 mL polypropylene tube. Exactly 2 volumes of absolute ethanol were added and the tubes were inverted gently several times until the DNA precipitated. The precipitated DNA strands were removed with a plastic spatula or pipette and transferred to a 1.5 mL microcentrifuge tube containing 100–200 pl TE buffer (10 mM Tris-HCl and 0.2 mM Na_2_EDTA, pH 7.5). The DNA was allowed to dissolve for 2 hours at 37°C before quantification. Genomic DNA from liver abscess pus samples for PCR was isolated using QIAamp DNA stool kit using manufacturer guidelines.

### 2.3. Primer Designing

*E. histolytica* specific primers were designed after Srivastava et al. from SINE2 [10]. EhSINE2 is highly abundant non-LTR, nonautonomous retrotransposon in* E. histolytica* genome [11]. Some of SINE2 copies show internal deletion, due to which the amplicon revealed size variation and multiple bands were observed near 350 bp. The nucleotide sequence of primer is as follows: F 5′-GTCAGAGACACCACATGAA-3′ and R 5′-CGAGACCCCTTAAAGAAACCC-3′ [10]. A set of PCR primers was designed to amplify the fragment of leptin receptor gene spanning the exon 6 locus of the gene carrying Q223R mutation. Primer sequences are F 5′-CCTGCTTTAAAAGCCTATCCAG-3′ and R 5′-AGTGTTAAGCAAAGTGAGATAAGC-3′. Primers sequences were bioinformatically analysed to ensure specificity using BLAST Programme of NCBI [12].

### 2.4. PCR-RFLP

**Figure 1:**
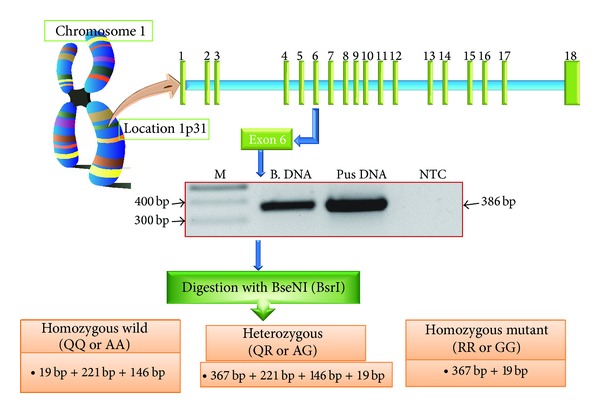
Schematic representation of the methodology followed for detection of Q223R mutation in leptin receptor gene. Digestion of PCR amplified product of 386 bp by restriction enzyme BseNI (BsrI) yields three bands of 221 bp + 146 bp + 19 bp in homozygous wild, four bands of 367 bp + 221 bp + 146 bp + 19 bp in heterozygous, and two bands of 367 bp + 19 bp in homozygous mutant. Allele “A” codes for glutamine and allele “G” codes for arginine in leptin receptor gene. Lane M = 100 bp Marker, lane B. DNA = human blood genomic DNA, lane pus DNA = ALA (amoebic liver abscess) pus DNA, and NTC = no template control.

**Figure 2:**
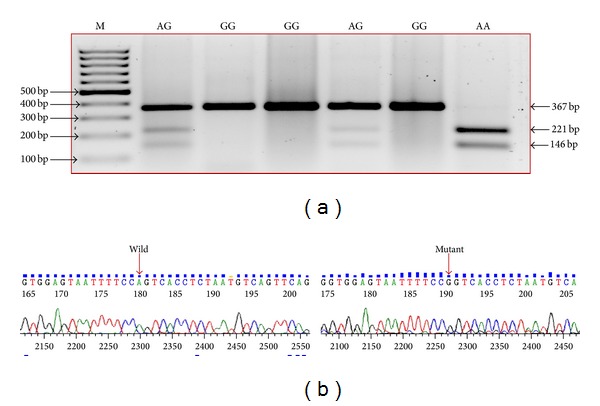
(a) PCR-RFLP analysis of Q223R SNP (rs1137101) from leptin receptor gene. Lane M = 100 bp Marker, lane AG, lane GG, and lane AA represent restriction enzyme digested PCR product of ALA pus DNA of different genotypes. After digestion with BseNI restriction enzyme, wild type AA is visible as two bands 221 bp, 146 bp and the third band of 19 bp is invisible due to smaller size. Heterozygous AG is visible as three bands 367 bp, 221 bp, 146 bp and 19 bp (invisible) whereas homozygous mutant GG is visible as bands of 221 bp, 146 bp and 19 bp (invisible). (b) Sequencing results of wild “A” and mutant “G” are detected by PCR-RFLP confirming the presence of SNP in DNA of ALA patients.

A total of 55 ALA samples along with 102 controls were subjected to PCR-RFLP analysis. DNA was amplified using leptin receptor specific primers. PCR was performed in a touch gene (Nugen Scientific, USA) machine. Thin walled 0.2 mL tubes were used for amplification. A typical PCR reaction (20 *μ*L) included 7.8 *μ*L of autoclaved milliQ water, 2 *μ*L of 10X PCR buffer with MgCl_2_ (containing 750 mM Tris-HCl (pH 8.8 at 25°C), 200 mM (NH_4_)_2_SO_4_, 0.1% Tween-20, 1.5 mM MgCl_2_), 2 *μ*L of dNTP mix (containing 2 mM of each dNTP), 2 *μ*L (20 pmol) of each primer forward as well as reverse, and 0.2 *μ*L of TaqDNA polymerase (5 U/*μ*L, MBI Fermentas, USA) and 2.0 *μ*L of template DNA. The amplification conditions were one cycle of 94°C for 5 min followed by 30 cycles of 94°C for 30 s, annealing 55°C for 1 min, extension at 72°C for 30 sec, and finally one cycle of 72°C for 10 min and finally held at to 4°C. Volume of template DNA used (2.0 *μ*L; ~50 ng) worked fine for PCR amplification. The sample containing all reagents except the template DNA was treated as the negative control. The size and integrity of the products were checked by electrophoresis. 10 *μ*L of the PCR product was run on a 0.8–1.2% agarose gel at 5 V/cm for an appropriate time period. Restriction enzyme BseNI was used to digest the PCR amplified product of 386 bp and the fragments generated upon digestion are represented in Figure 1. Restriction enzyme BseNI digests only when the sequence reads nucleotide A at the locus. Thus digestion of 386 bp PCR product yielded three bands of 221 + 146 + 19 bp in case of homozygous (AA, assuming A as wild allele) wild and two bands of 367 + 19 bp in case of homozygous mutant (GG) (Figure 1). As expected, the digestion of heterozygous mutant yielded four bands of 367 + 221 + 146 + 19 bp as shown in a representative gel. All bands except 19 bp were visible on 1.5% agarose gel. Sequencing of mutated fragment confirmed the presence of mutation in Indian population (Figures 2(a) and 2(b)).

### 2.5. Statistical Analysis

Data was evaluated by SPSS software version 12 using standard contingency *χ* ^2^ tests or Fisher's exact test for calculating the differences in genotype frequency between cases and controls. A two-tailed *P* value <0.05 was considered significant. Multiple comparisons were done using one way ANOVA based on the conservative Bonferroni correction. The significance level of *α* = 0.05 was chosen for all sets.

## 3. Results and Discussion

**Figure 3:**
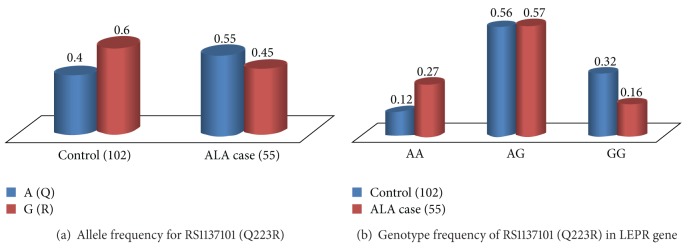
Allele and genotype frequencies of SNP Q223R (rs1137101) in* LEPR* gene in amoebic liver abscess patients and control samples of North India. (a) Distribution of allele frequency. Allele “A” codes for glutamine whereas allele “G” codes for arginine amino acid in* LEPR.* (b) Distribution of genotype frequency.

Out of 55 collected samples, 54 samples gave positive result with PCR conducted with* E. histolytica* specific primers accounting for 98% efficiency in diagnosis. We assessed the association of the SNP with a number of different diseases related outcomes and for possible confounding variables. Genotype and allele frequencies for SNP rs1137101 (Q223R) in* LEPR* gene of ALA cases were stratified by phenotypic subgroups and represented in Table 2. Genotype and phenotype profiling of ALA patients studied here revealed that gender, age, and alcoholism are other important risk factors for amoebic liver abscess. Frequency of allele G was calculated and is represented in Figure 3(a). We did not observe significant difference in allele frequency of “G” among control and ALA patients. However, the distribution of genotype frequency followed the following pattern AA < GG < AG in control and GG < AA < AG in ALA patients of North India (Figure 3(b)). Fisher exact test was performed to check the association of mutation Q223R with ALA using SPSS version 12 software. Our results showed that heterozygous mutant (QQ versus QR, *P* = 0.049) and homozygous mutant (QQ versus RR, *P* = 0.004) were significantly associated with amoebic liver abscess when compared with homozygous wild (QQ) (Table 3). Mutation Q223R in leptin receptor gene is very important as it increases the susceptibility of* E. histolytica* infection in malnourished children. Malnutrition represents a significant health problem in the developing world including India and growing body of evidence has indicated an epidemiological connection between susceptibility to infection and malnutrition. The leptin levels in malnourished children have been reported to be lower than the well-nourished ones with a concomitant suppression of inflammatory responses [13, 14]. Two recent studies had explored the link between malnutrition, leptin signaling, and susceptibility to amebic infection. The first study by Duggal et al. involved the prospective observation of a cohort of 185 Bangladeshi children by household visits every other day over a period of nine years. During this study period, 90 percent of the children enrolled had at least one bout of* E. histolytica* infection. The children were also tested for polymorphisms in their leptin and leptin receptor genes. They found that mutation Q223R increased susceptibility to intestinal infection by* E. histolytica* depending on the presence of allele “G” in homozygous or heterozygous state [5].

The second study by Guo et al. showed that mice lacking the functional leptin receptor developed devastating mucosal destruction after* E. histolytica* infection [15]. Leptin-mediated resistance to amebiasis is via its action on intestinal epithelium rather than hematopoietic cells or the brain and requires leptin receptor signaling through STAT3 [15]. The in vitro studies have shown that the Q223R polymorphism in leptin receptor attenuates leptin-dependent STAT3 activation to that of the wild-type (WT) receptor and it is the leptin regulation of host apoptotic genes TRIB1 and suppressor of cytokine signaling 3 (SOCS3) via STAT3 which is responsible for protection [16]. A recent study in* E. histolytica* infected mice (223R mice compared to Q223 mice) has shown that the majority of leptin-linked differentially regulated genes were involved in apoptosis, cellular proliferation, or recruitment of hematopoietic cells. The differential regulation of these genes suggests that the Q223R polymorphism attenuates the ability of* LEPR* stimulation to protect cells against amebic killing and/or apoptosis [17]. Similarly our study also shows that most of the ALA patients had “AG” genotype and allele “G” is associated with ALA patients. The presence of allele “G” is an important risk factor in Indian population.

## 4. Conclusion

Our study concludes that the mutation Q223R is associated with susceptibility to* E. histolytica* infection in North Indian population but large population based studies are needed to confirm our observation in Indian population. The frequency of allele “G” is higher in Indian population than that of allele “A.” The worldwide distribution of allele “G” in Q223R mutation shows that it is more prevalent in Asian and African subcontinents whereas allele “A” is more predominant in European population.

## Figures and Tables

**Table 1:** Demographic and clinical details of amoebic liver abscess patients (*n* = 55).

(1) Sex: *n* male (%)/female (%)	48 (87.2)/7 (12.7)
(2) Age at diagnosis: mean (SD)	43 (13.9)
15–30: *n* (%)	9 (16.12)
31–above: *n* (%)	46 (83.87)
(3) Duration of disease (range)	7–20 days
(4) Alcoholic: *n* (%)	
Yes	36 (65.45)
No	16 (29.0)
Ex	3 (5.45)
(5) Location of abscess: *n* (%)	
Right lobe of liver	51 (92.70)
Left lobe of liver	4 (7.3)
(6) No. of abscesses (single/multiple): *n* (%)	41/14 (75/25)
(7) Whether first aspiration (yes/no): *n* (%)	55/0 (100/0)
(8) Drugs for treatment	Metronidazole and ciprofloxacin
(9) Ethnicity	Aryan
(10) Location	North India

**Table 2:** Genotype and allele frequencies for SNP rs1137101 (Q223R) in *LEPR* gene of ALA cases stratified by phenotypic subgroups (*n* = 55).

SNP	SNP rs1137101 (Q223R) in *LEPR* gene			
Genotype	AA (15)	AG (31)	GG (9)	Total (55)
(1) Sex				
Male	10	30	8	48
Female	5	1	1	7
(2) Age at diagnosis (Yr)				
15–30	5	4	0	9
31–above	10	27	9	46
(3) Alcoholic				
Yes	11	19	6	36
No	4	10	2	16
Ex	0	2	1	3
(4) No. of abscesses				
Single	8	27	6	41
Multiple	7	4	3	14

**Table 3:** Association of leptin receptor gene (*LEPR*) polymorphism at Q223R locus with amoebic liver abscess. ALA case (*n* = 55), Control (*n* = 102), Q (glutamine) = A (wild), and R (arginine) = G (mutant). *P* value = or <0.05 was considered significant. ∗Refers to significant *P* values.

rs1137101	*N* = ALA (control)	OR	95% CI	*P* value
QQ versus QR	15 (12) : 31 (57)	0.435	0.181–1.045	0.049∗
QQ versus RR	15 (12) : 9 (33)	0.218	0.076–0.629	0.004∗
QR versus RR	31 (57) : 9 (33)	0.501	0.213–1.182	0.08
